# Nano Milk Protein-Mucilage Complexes: Characterization and Anticancer Effect

**DOI:** 10.3390/molecules26216372

**Published:** 2021-10-21

**Authors:** Ahmed Ali Abd El-Maksoud, Amal I. A. Makhlouf, Ammar B. Altemimi, Ismail H. Abd El-Ghany, Amr Nassrallah, Francesco Cacciola, Tarek Gamal Abedelmaksoud

**Affiliations:** 1 Dairy Science Department, Faculty of Agriculture, Cairo University, Giza 12613, Egypt; ismailabdelghany@gmail.com; 2 Pharmaceutics and Industrial Pharmacy Department, Faculty of Pharmacy, Cairo University, Cairo 12411, Egypt; amal.makhlouf@pharma.cu.edu.eg; 3 Food Science Department, College of Agriculture, University of Basrah, Basrah 61004, Iraq; ammar.ramddan@uobasrah.edu.iq; 4 Biochemistry Department, Faculty of Agriculture, Cairo University, Giza 12613, Egypt; amotagly@cu.edu.eg; 5 Department of Biomedical and Dental Sciences and Morphofunctional Imaging, University of Messina, 98125 Messina, Italy; 6 Food Science Department, Faculty of Agriculture, Cairo University, Giza 12613, Egypt; tareekgamal_88@agr.cu.edu.eg

**Keywords:** milk proteins, Isabgol husk mucilage, Nabeq mucilage, milk proteins mucilage complexes, anticancer activity

## Abstract

The anticancer activity of natural compounds has recently attracted multidisciplinary research. In this study, the complexation of milk proteins (MP) with Isabgol husk mucilage (IHM) and *Ziziphus spina-christi* mucilage (NabM) was investigated. In this context, the physicochemical properties of milk protein mucilage complexes (MPMC) including pH, Carr’s index, water solubility, and water absorption indices were measured, and the flow behavior was studied. In addition, the amino acid profile, protein digestibility, and phenolic and flavonoids content of MPMC were explored, and the microstructure of the complexes was visualized using transmission electron microscopy. The antioxidant and anticancer potencies of MPMC against two cancerous cell lines, human liver cancer HEPG-2 and breast cancer MCF-7, in comparison with two normal cell lines, namely, Bj-1 and MCF-12F, were tested using neutral red uptake assay. The results revealed that MPMC had scavenging activity against DPPH, ABTS, and HS radicals. Moreover, MPMC has the potential to prevent DNA damage induced by oxidative stress in Type-Fenton’s reaction. The results of the neutral red assay showed significant growth inhibition of both HEPG-2, MCF-7, whereas no significant cytotoxic effect was detected against Bj-1 and MCF-12F. RT-qPCR results indicated MPMC stimulated apoptosis as revealed by the upregulation of the pro-apoptosis gene markers Casepase-3, p53, Bax. Meanwhile, the anti-apoptosis Bcl-2 gene was downregulated. However, no significant difference was observed in normal cell lines treated with MPMC. In conclusion, MPMC can be considered as a promising anticancer entity that can be used in the development of novel cancer therapeutics with comparable activity and minimal side effects compared to conventional cancer chemotherapies.

## 1. Introduction

Cancer is the primary cause of mortality around the world with approximately 10 million deaths in 2020 [1]. Early diagnosis and the development of novel therapeutics is the only hope to defeat cancer. Conventional cancer treatments such as chemotherapy and radiation are relatively expensive and accompanied by significant side effects.

Therefore, scientific and research interest is tending to the utilization of natural compounds (i.e., proteins, polysaccharides, and polyphenols) from their sources that have anticarcinogenic potential as they are considered to have less toxic side effects compared to conventional treatments.

Polysaccharides from plant sources have recently received increasing attention due to their bioactive properties [2,3]. Numerous studies have been interested in isolating bioactive polysaccharides and polyphenols from natural plant sources such as fruits, vegetables, cereals, and herbs due to their beneficial pharmacological effects [4].

*Plantago ovata* (Psyllium or Isabgol) and *Ziziphus spina-christi* (Nabeq or Sidr) are known sources of bioactive polysaccharides. The IHM and NabM were selected as promising bioactive materials. Previous studies indicated that IHM and NabM complexes with milk proteins improved liver function and diminished the risk of cardiovascular diseases [5]. Isabgol husk has been proven to be effective for the treatment of diarrhea, constipation, ulcerative colitis, irritable bowel syndrome, hypercholesterolemia, and diabetes. Moreover, the anticancer effect of isabgol husk against colorectal cancer was attributed to its fiber content, which acts to decrease its effects by reducing transit time. This will lead to reducing bile metabolism by the gut microflora, dilution of the bile acids by stool bulking, alteration of microbial bile acids metabolism due to fiber fermentation, reduction of the pH and production of short chains fatty acids, or by direct binding to the bile acids and hence preventing their metabolism [6]. The main biologically active components of *Ziziphus spina-christi* are vitamin C, phenolics, flavonoids, and triterpenic acids. Its bioactivities include anticancer, antibacterial, antidiabetic, antiproliferative, and antioxidant activities [7]. Isabgol husk and Nabeq fruits are used for the production of Isabgol husk mucilage (IHM) and Nabeq mucilage (NabM), respectively [5].

On the other hand, protein-based therapies for cancer have gained increasing interest due to key features such as low cytotoxicity, strong specificity, and ease of modification [8]. In addition to providing the nutritional needs of essential amino acids, milk proteins are among the biological macromolecules that have many functional properties such as antioxidant, immunomodulatory, antidiabetic, antimicrobial, and anticancer properties [9,10]. For instance, the anticancer activity of dairy-derived peptides (i.e., β-Casomorphins isolated from β-casein and α1-casein fragments 90–95 and 90–96 [Arg90-Tyr-Leu-Gly-Tyr-Leu95-(Glu96)]) identified in bovine milk support the assumption that milk proteins are not only of nutritious value but also have the potential for cancer prevention and treatment [11,12].

Complexation between milk proteins and polysaccharides, such as milk proteins-chitosan complex, was previously produced as an attempt to enhance the functional properties of their natural components [13]. Due to the availability and ease of preparation, such complexes have potential to be used in the development of less expensive anticancer therapies. Such therapeutics will be less expensive and show comparable activity to the currently used chemotherapeutics.

Non-covalent binding through hydrophobic and electrostatic interactions are the primary factors in the association of milk proteins with polysaccharides [14]. Moreover, proteins show surprising resilience towards interaction with other components at the range of pH from 2 to 11. At a specific pH, the surface reactivity of protein increases through the unfolding of protein structure [15].

In this context, our group has recently focused on the functional role of milk protein complexes on human health. However, milk protein complexes with IHM or NabM, have exhibited antihyperlipidemic and liver-protective properties [9]. Thus, based on previous studies, it was postulated that milk protein-polysaccharide complexes might have interesting anticancer activity. Additionally, no publications had conducted a study of the anticancer activity of milk proteins complexes with IHM and NabM. Therefore, the main objectives of this study were to characterize the physicochemical properties of the newly produced milk protein mucilage complexes including, amino acid profile and functional properties of these complexes. In addition, the anticancer activity of milk protein complexes with IHM and NabM mucilage against two human cancerous cells (MCF7 and HEPG2) was investigated in comparison with non-cancerous cell lines (Bj-1 and MCF-12F). We also shed light on the mode of action of the produced milk proteins mucilage complexes.

## 2. Results and Discussion

### 2.1. Fourier-Transform Infrared (FTIR) Spectroscopy

**Figure 1:**
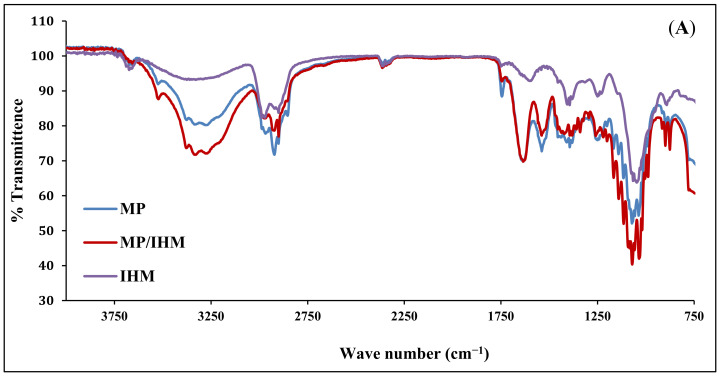

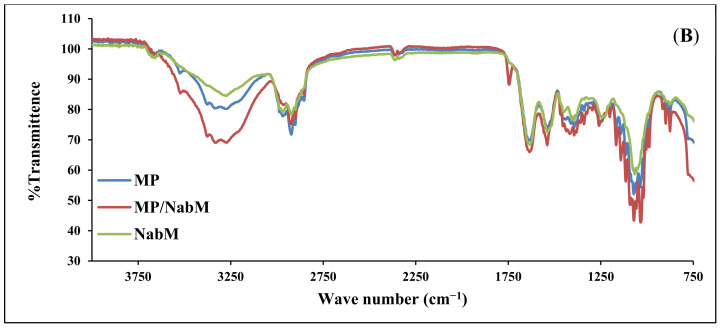
(**A**) Fourier-transform infrared spectra of MP, IHM, and MP/PHM. (**B**) Fourier-transform infrared spectra of MP, IHM, and MP/NabM. NabM: Nabeq mucilage, IHM; Isabgol husk mucilage, MP: milk proteins concentrate, MP/IHM: milk proteins/Isabgol husk mucilage complex, MP/NabM: milk proteins/Nabeq mucilage complex.

The infrared spectra of IHM, MP, and MP/IHM are illustrated in Figure 1A. The spectrum of IHM showed the characteristic band of arabinoxylans at 100–1200 cm^−1^ and the bands for the amide I and amide II groups at 1550 and 1650 cm^−1^, respectively. The MP spectrum shows bands at 1700–1600 and 1200–900 cm^−1^ corresponding to amide I (mainly C=O stretching of proteins) and milk carbohydrates, respectively [16]. The aforementioned peaks of IHM and MP were reserved in the spectrum of the IHM/MP complex. Similarly, the characteristic bands of galacturonic acid in NabM were maintained in the spectrum of the NabM/MP complex (Figure 1B). This would indicate the absence of chemical interaction between polysaccharide mucilage and MP and thus confirm the proposed mechanism of electrostatic attraction between MP and polysaccharides (IHM and NabM). Such observations are in line with those of Vukic et al. who reported the complex formation between whey milk proteins and pectin through electrostatic and hydrophobic interactions which resulted in good consistency of the complex [17].

### 2.2. Physicochemical and Functional Properties of MP and MPMC

The values of bulk density (BD), tapped density (TD), Carr’s Index, and pH measurements are listed in Table 1. BD and TD of MP were higher than IHM, and IHM had the lowest bulk and tapped density of the tested samples. Similarly, MP/IHM complex had significantly (*p* < 0.05) lower bulk and tapped density compared to MP/NabM. The obtained results indicated that the distribution and solubility of MP have been improved by complexation with the IHM. Carr’s index is frequently used as an indication of the flowability of powders. The results in Table 1 show that Carr’s index of MPMC was significantly lower than IHM which indicated that NabM improved the flowability of MP. This was probably due to the lower Carr’s Index value of MP (30.6 ± 0.10%) compared to IHM and NabM (57.58 ± 0.14 and 53.97 ± 0.11%, respectively) which are viscous. IHM is alkaline (7.79 pH) while MP and the complexes with polysaccharide mucilage were slightly acidic which came in accordance with previously reported results [18].

WSI and WAI are important functional features in food technology. Table 1 shows that WSI and WAI of MP significantly increased after complexation with IHM and NabM. This was most likely because Isabgol-based materials are highly water soluble with a thickening effect following the addition of adequate amounts of water [19]. Additionally, Qaisrani et al. reported that arabinoxylans of Isabgol husk have a water holding capacity up to ten-fold that of their dry weight [20]. Moreover, MP/IHM has higher WSI and WAI than MP/NabM since IHM has higher WSI and WAI than NabM. Additionally, the increase of WSI % for MPMC compared to MP led to an increase in the dispersibility of the target active components when utilizing them in therapeutics issues [21]. This was clear in the anticancer results which demonstrated that these complexes have a cytotoxic effect than MP.

### 2.3. Bioactive Components

#### 2.3.1. Phenolic Compounds of PHM and NabM

**Figure 2:**
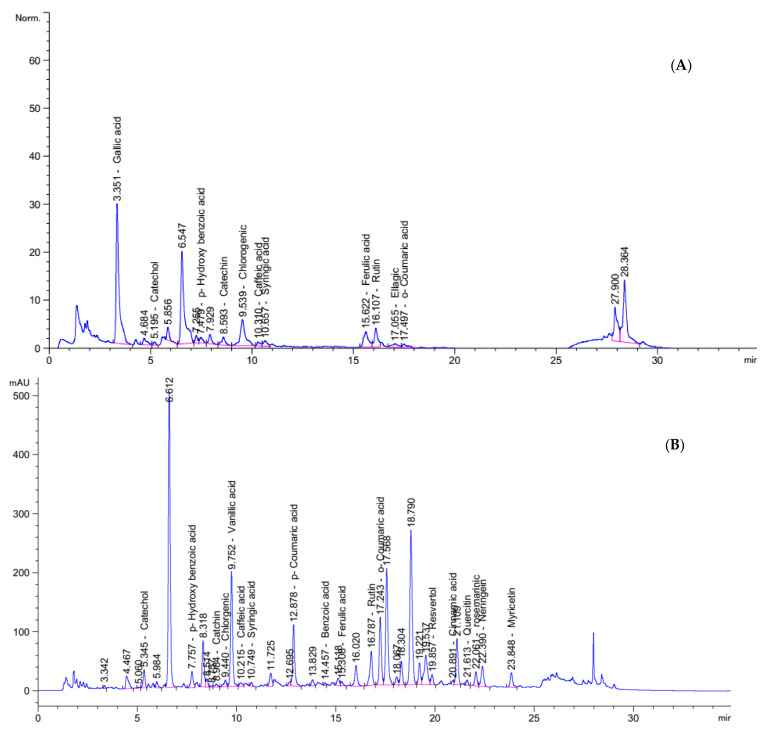
HPLC analysis of polyphenolic profiles of (**A**) IHM and (**B**) NabM.

Food contains very important bioactive compounds (i.e., phenolic and flavonoids) which act as antioxidant and anticancer potencies. HPLC analysis confirmed and quantified the presence of many phenolic compounds in Nab and IHM as demonstrated in Figure 2. The results show the high content of various biologically active compounds such as gallic acid (177.96 mg/Kg), catechol (13.87 mg/Kg) p-Hydroxy benzoic acid (24.02 mg/Kg), Catechin (5.93 mg/Kg) and rutin (123.70 mg/Kg) in PHM extract and Catechol (410.72 mg/Kg) p-Hydroxy benzoic acid (426.71 mg/Kg) Chlorgenic (72.05 mg/Kg) Rutin (1750.57 mg/Kg), and rosemarinic (1771.72 mg/Kg) in NabM (Figure 2A,B). The content of these compounds is very important for health benefits. The anticancer effect of phenolic compounds was previously reported. This effect was mainly attributed to the antioxidant effect of these compounds.

#### 2.3.2. Total Phenolic, Flavonoid, and Antioxidant Activity of MP and MPMC

**Figure 3:**
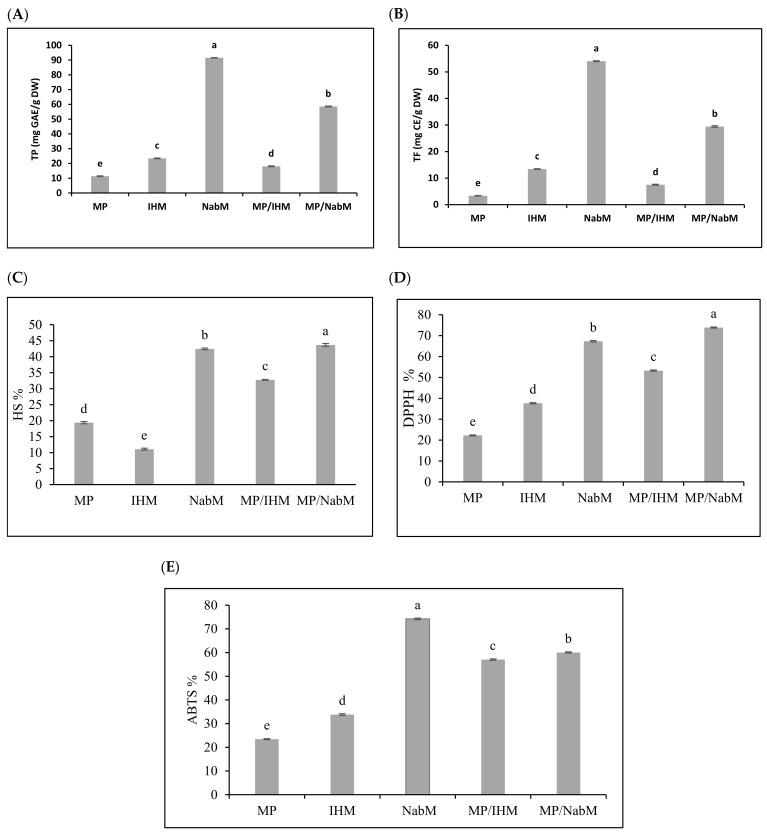
Total phenolic and total flavonoid content (**A**,**B**) and antioxidant activity (**C**–**E**) of MP, IHM, NabM, and MPMC. MP: milk proteins concentrate; IHM: Isabgol husk mucilage; NabM: Nabeq mucilage; TPC: total phenolic content; GAE: gallic acid equivalent; TF: total flavonoid; CE: catechin equivalent; DW: dry wight; DPPH: 2,2-diphenyl-1-picrylhydrazyl; ABTS: 2,2′-azino-bis (3-ethylbenzothiazoline-6-sulphonic acid; HS: hydroxyl scavenging; Values are means ± SD (n = 3). Measurements with different letters (a, b, c, d, and e) are significantly different (*p* < 0.05).

Several reports have indicated that compounds that exhibit antioxidant properties mostly display anticancer activity [22]. Total phenolic (TP) and total flavonoid (TF) content were determined in NabM, IHM, MP, and their complexes (Figure 3A,B). Results indicated that TP and TF were higher in NabM and MP/NabM compared to IHM and MP/IHM. However, TP and TF contents were significantly lower in MP compared to MPC. These results came in agreement with data reported by Singh et al. [23] who found that TP and TF of NabM were 1.6 GAE mg/100 g dry weight and 47 mg CE/100 g dry weight, respectively. MP/NabM and MP/IHM complexes showed a significant increase by 309% and 59% in TP and 476% and 123% in TF compared to MP, respectively. Conjugation of MP with IHM and NabM led to the loss of 23% and 36% of TPC corresponding IHM and NabM, respectively. TF was reduced by 44% in IHM and 46% in NabM when complexed with MP. This was most probably due to the low TP and TF of MP.

The potential radical scavenging activity of MP/IHM and MP/NabM compared to MP using DPPH, ABTS, and HS is illustrated in Figure 3C–E. The data obtained from the three antioxidants activity had a similar trend indicating that NabM displayed higher antioxidant activity than IHM and this is in the context of the results of phenolics and flavonoids contents. The antioxidant capacity of MP was increased after complexing with NabM and IHM. With the exception in ABTS assay, the antioxidant activity of NabM increased by conjugation with MP. The antioxidant activity indicated that MPMC had higher antioxidant activity compared to MP. Our hypothesis points to a possible interaction between MP and polysaccharides that lead to gaining the potential antioxidant activity as described by Li et al. [24]. It has been previously reported that there is positive correlation between the scavenging capacity and phenolic/flavonoids content [5]. However, we cannot exclude that new bioactive peptides enriched with sulfur-containing amino acids are produced, explaining the high antioxidant activity of MPMC compared to the uncomplexed components. Moreover, the small peptides are released in whey fractions influencing the high antioxidant properties [4]. Finally, phenolic and flavonoid contents affect different cancer cells. Additionally, during in vitro cell culture assays, these activities and mechanisms were observed. Antioxidant and anticancer activities of phenolics are attributed to double bonds and hydroxyl substitutions on the aromatic rings [25].

### 2.4. Amino Acid Profile of MP and MPMC

**Figure 4:**
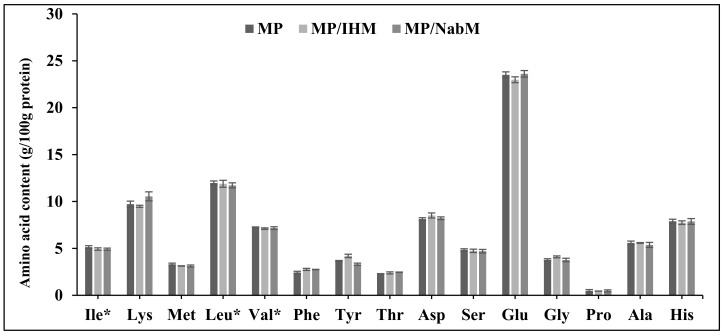
Amino acid profile of MP, MP/IHM, and MP/NabM. MP: milk proteins concentrate, MP/IHM: milk proteins/Isabgol husk mucilage complex and MP/NabM: milk proteins/Nabeq mucilage complex.* essential amino acids.

Amino acid profiles of MP, NabM, MP/IHM, and MP/NabM are illustrated in Figure 4. There were no significant differences in amino acid profiles of MP and its complexes with IHM and NabM. This indicated that the non-covalent interaction of MP with polysaccharide mucilage did not change the composition of milk proteins. The results show the high content of glutamic acid in MP and MPMC (Figure 4) [26].

### 2.5. Rheological Properties of MP and MPMCs

#### 2.5.1. Apparent Viscosity

Figure S1 shows the viscosity of protein samples at different rpm values. IHM and NabM had high viscosity at slow rotation, but the viscosity decreased significantly as the rotation speed increased. These findings were in agreement with Chen and Che who recorded that the apparent viscosity of water-soluble mucilage of green laver (*Monostroma nitidium*) decreased by increasing the shear rate [27].

The results in Figure S1 also demonstrate that the complexation of MP with IHM and NabM resulted in a significant increase (*p* ≤ 0.05) in the apparent viscosity of MP. This could be attributed to the unique properties of IHM (hydrocolloid material) which has a viscosity-enhancing effect [28].

#### 2.5.2. Flow Behavior

The flow behavior (shear stress/shear rate curves) of milk proteins and their complexes is illustrated in Figure S2. With the increase in shear rate, shear stress increased. Transforming the shear stress and shear rate data to the power-law equation, the values of the flow consistency index (K) and flow behavior index (n) are shown in Table S1. IHM and MP/IHM had the highest flow consistency index. This might be due to the high viscosity of water-soluble mucilage of IHM due to its high capacity for holding water and gelling in an aqueous medium. The results also demonstrated that IHM, NabM, and MP/IHM had shear-thinning behavior (non-Newtonian fluids) with a low n value ranging from 0.25 to 0.64. These results came in accordance with Thanatcha and Pranee who studied the physicochemical properties of *Ziziphus mauritiana* [29]. On the other hand, the n value of MP was approximately 1 (0.99) indicating Newtonian flow. Similarly, the n value for the MP/NabM complex was 0.95 as shown in Table S1.

### 2.6. Differential Scanning Calorimetry of MP and MPMC

Differential scanning calorimetry was used to investigate the thermal stability of MP and its complexes with IHM and NabM. The thermograms of MP and MPMC are illustrated in Figure S3, and the DSC parameters are provided in Table 2. It was observed that there was no clear endothermic peak on MP thermogram, but there was a prominent endothermic peak for MP/IHM and a broad endothermic peak for MP/NabM. This is probably due to the gradual elimination of free water, the melting point of NabM, slow gelation, and protein denaturation [30].

It was also noted that MPMC was more heat-stable than uncomplexed protein as observed by the low denaturation temperature of MP (101.44 °C) compared to MP/IHM and MP/NabM (148.24 and 121.4 °C, respectively) as shown in Table 2. Moreover, the degradation temperature of MP was recorded to be 302.3 °C, whereas no degradation of MPMC was observed with a temperature increase up to 350 °C. These findings were in agreement with several studies, which found that the thermal stability of milk proteins increased by the interaction with phenolic compounds which prevented protein aggregation [2,31].

The heat that passes into or out of the system during a reaction is the enthalpy (ΔH). The higher enthalpy of MP/IHM and MP/NabM compared to that of the uncomplexed MP could be due to the potential chemical reaction between milk proteins (whey and casein) and between milk proteins and polysaccharides at high temperature (Maillard reaction) [31].

### 2.7. Transmission Electron Microscopy (TEM) of MP and MPMC

**Figure 5:**
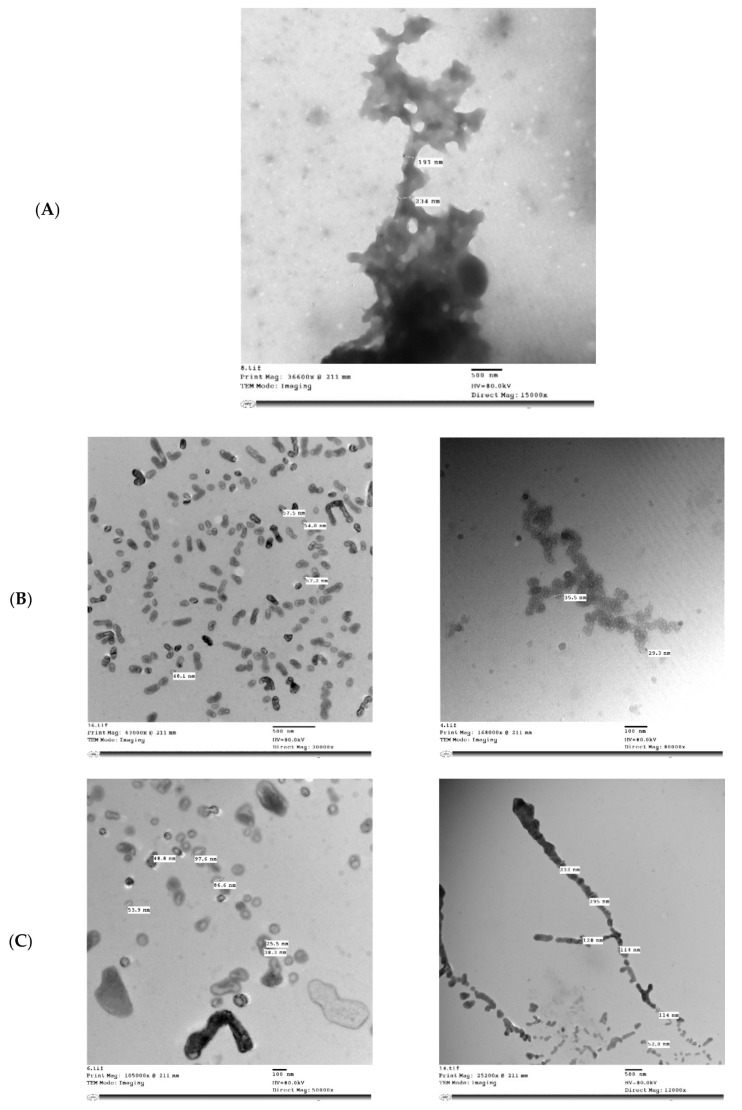

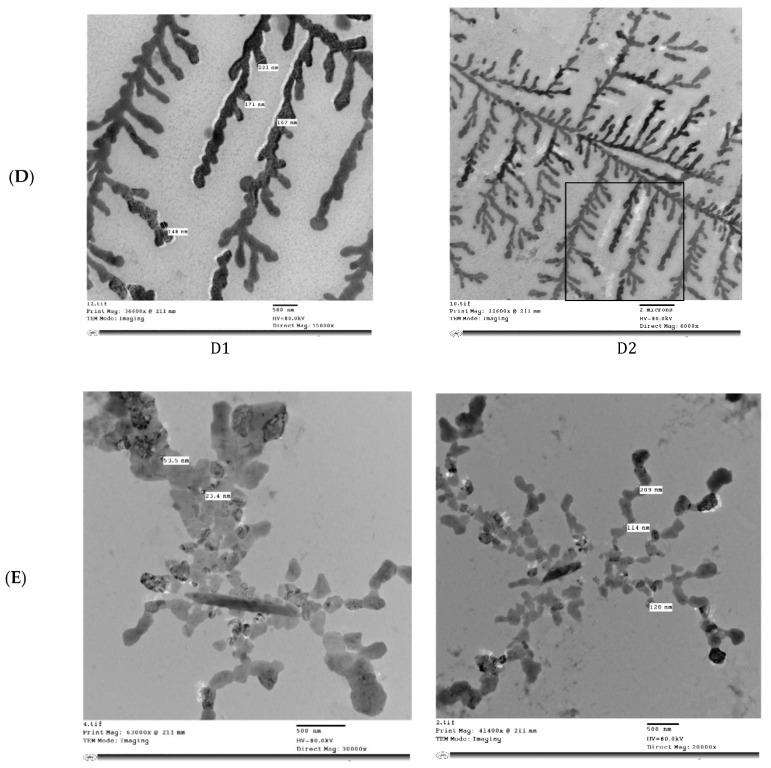
Transmission electron microscopy images of (**A**) MP, (**B**) IHM, (**C**) NabM, (**D**) MP/IHM, and (**E**) MP/NabM. Panel D2 shows the original micrograph; the boxed region indicates the area enlarged in panel D1. MP: milk protein concentrate; MP/NabM: milk protein–Nabeq mucilage complex; MP/IHM: milk protein–Isabgol husk mucilage complex.

Figure 5 shows the microstructure of IHM, NabM, MP, and MPMC. It was found that the particle size of MPMC was in the nano range (114–250 nm), and it was smaller than the particle size of MP (191–300 nm). Additionally, TEM of MPMC (Figure 5D,E) revealed the branched pattern of the formed complexes. This could be attributed to the electrostatic attraction between milk proteins and polysaccharides Goh, et al. [32]. Moreover, the larger branched structure of MP/IHM compared to MP/NabM might be responsible for its higher viscosity as shown in Figure S1.

### 2.8. Biological Studies on MP and MPMC

#### 2.8.1. Anticancer Activity of MP and MPMC

**Figure 6:**
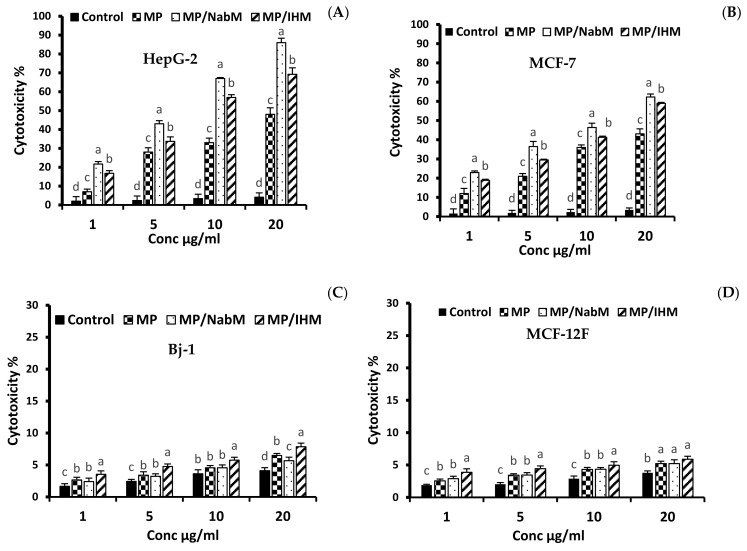
The anticancer activity of MP, MP/NabM, and MP/IHM on HepG-2 (**A**), MCF-7 (**B**), Bj-1 (**C**), and MCF-12F (**D**) cell lines at different concentrations (1, 5, 10, and 20). The cytotoxicity was evaluated calorimetrically by neutral red uptake assay. MP: milk proteins concentrate; MP/NabM: milk proteins/Nabeq mucilage complex; MP/IHM: milk proteins/Isabgol husk mucilage complex. Three replicates of each treatment were analyzed. Measurements with different letters (a, b, c and d) are significantly different (*p* < 0.05).

The cytotoxic effect of various concentrations (1, 5, 10, and 20 µg/mL) of MP and MPMC on HepG-2 and MCF-7 cells compared to MCF-12F and Bj-1 normal cells is illustrated in Figure 6. MPMC exhibited anticancer effect on HepG-2 and MCF-7 cells Figure 6A,B, respectively. The dose-responsive curve exhibited a reduction in cell viability in response to higher concentrations. On the other hand, no significant cytotoxic effect of MP and MPMC on Bj-1 and MCF-12F normal cells was observed (Figure 6C,D). Our results also indicated that MP/NabM had higher anticancer activity against HepG-2 and MCF-7 cancer cell lines, with IC_50_ value 5.13 and 10.07 µg/mL, respectively. Additionally, MP/IHM exhibited moderate cytotoxic effect against cancer cell lines compared to respective controls. Altogether, our cytotoxic data suggest that complexing MP with IHM and NabM significantly enhances the anticancer activity compared to uncomplexed milk proteins, as shown in Table 3. It was also evident that HepG-2 cells were more sensitive to MP and MPMC compared to MCF-7 cells as manifested by the lower values of IC_50_.

The anticancer effect of milk protein-derived antitumor peptides has been previously reported. In this context, Jeong and Hong [33] observed that trypsin hydrolysates of α- lactalbumin had a cytotoxic effect on human bone cancer SJSA-1, human colorectal cancer HCT 116, and human gastric cancer NCI-N87 cell lines. It was found that α-lactalbumin, interact with cell surface modulators and alter cell growth rate, intracellular calcium, and the calcium transport rate [34]. It is worth noting that bovine lactoferrin induced apoptosis in MCF7 cells in a dose-dependent manner [35]. Moreover, the casein and whey protein fractions of milk showed antitumor effects in an animal model as well as in vitro antiproliferative activity [36]. In addition, Elzoghby et al. [37] reported that casein nanoparticles can be used to encapsulate the hydrophobic anticancer drug, flutamide, to control the drug release, improve its anti-tumor activity, and decrease its hepatotoxicity.

The anticancer effect of IHM and NabM was previously reported [6,38]. The cytotoxic effect of MP and MPMC might be attributed to the presence of amino acids such as glutamine, arginine, and cysteine which are reported to exhibit anticancer activity against colorectal cancer [39]. These results are in agreement with the high content of glutamic acid of MPMC (Figure 5).

#### 2.8.2. Determination of p53, Bax, Caspase-3, and Bcl-2 Proteins Level

**Figure 7:**
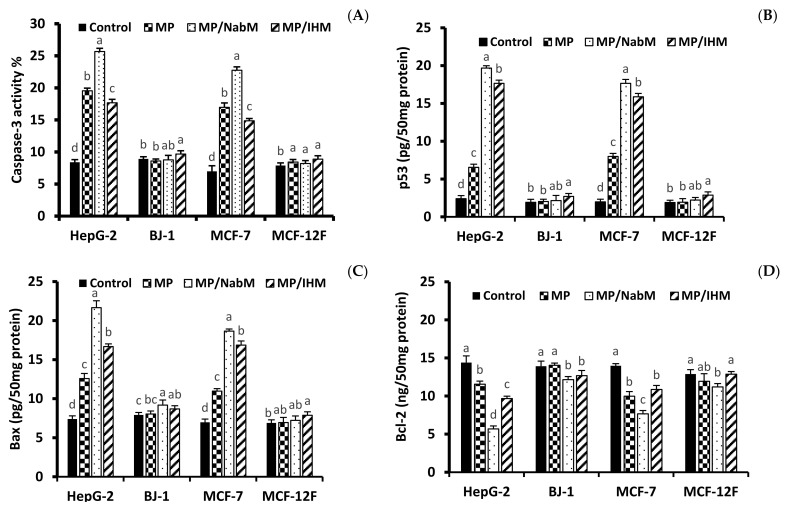
Protein levels of apoptosis biomarker. The level of Caspase-3 (**A**), p53 (**B**), Bax (**C**), and Bcl-2 (**D**) in HepG-2, MCF-7, Bj-1, and MCF-12F treated with or without the IC_50_ of MP, MP/NabM, and MP/IHM. MP: milk proteins concentrate; MP/NabM: milk proteins/Nabeq mucilage complex; MP/IHM: milk proteins/Isabgol husk mucilage complex. Data are average of triplicates. Measurements with different letters (a, b, c and d) are significantly different (*p* < 0.05).

In order to investigate the cytotoxicity mechanism of MPMC, the level of apoptosis marker proteins p53, Bax, Caspase-3, and Bcl-2 was determined in HepG-2, MCF-7, Bj-1, and MCF-12F cell lines treated with IC_50_ of MPMC (Figure 7). The level of pro-and anti-apoptotic markers is associated with apoptosis induction in cancer cell lines [40,41]. Results indicated that MP/NabM and MP/IHM enhanced apoptosis in HepG-2 and MCF-7, but not in MCF-12F and Bj-1 cell lines. The level of caspase-3, p53, and Bax significantly increased compared to respective controls (Figure 7), whereas Bcl-2 protein was reduced in all treatments. Interestingly, MP also showed a significant alteration in apoptosis protein markers. This was consistent with the results of anticancer study and could be referred to the cytotoxic effect of a number of milk proteins-derived peptides as discussed above (Section 2.8.1).

#### 2.8.3. DNA Damage Protection by MPMC against Oxidative Cleavage of RNH1 Plasmid

**Figure 8:**
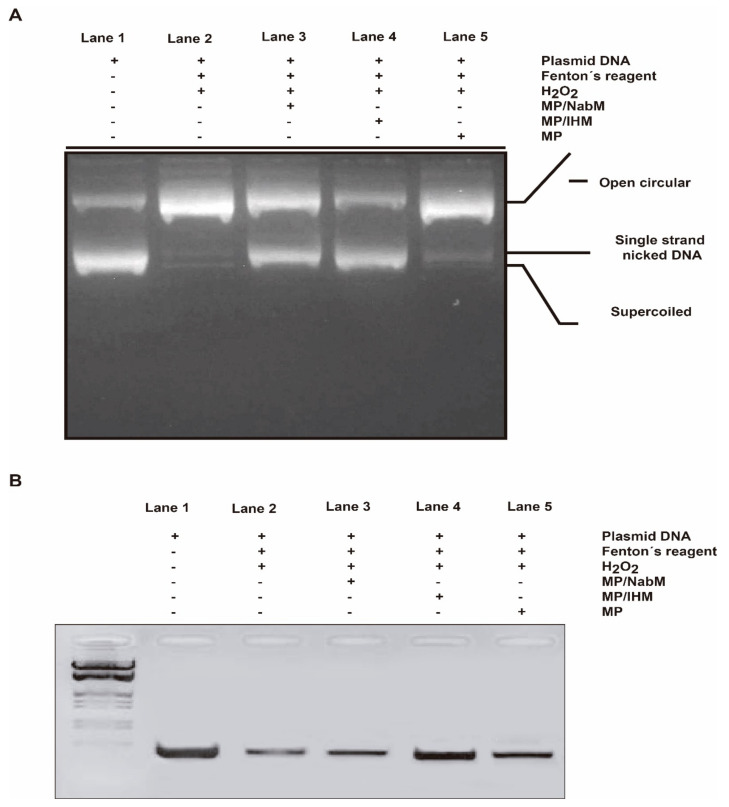
DNA damage protection by MP/NabM and MP/IHM. (**A**) The protective capacity MP/NabM, MP/IHM, and MP against RNH1 plasmid DNA damage induced by Fenton’s reagent. (**B**) The protective capacity MP/NabM, MP/IHM, and MP against genomic DNA damage. Lane 1: DNA control, lane 2: DNA treated with Fenton’s reagent, lane 3: DNA treated with Fenton’s reagent plus MP/NabM, lane 4: DNA treated with Fenton’s reagent plus MP/IHM, and lane 5: DNA treated with Fenton’s reagent plus MP. MP: milk proteins concentrate; MP/NabM: milk proteins/Nabeq mucilage complex; MP/IHM: milk proteins/Isabgol husk mucilage complex.

The *RNH1* plasmid DNA has three forms on agarose gel electrophoresis ordered from top to bottom as open circular, linear, and supercoiled circular DNA. The oxidative stress by Fenton’s reagent induced DNA damage. Subsequently, the level of DNA forms is altered. However, open circular and linear forms indicate DNA damage and supercoiled circular DNA indicates more protection potential. In this assay, we tested whether MP and MPMC at their IC_50_ can protect against the degradation of supercoiled circular form in response to oxidative stress. As a result, both MP/NabM and MP/IHM showed high protection capacity similar to DNA control (lanes 3 and 4, Figure 8A). On the other hand, no significant difference was observed in DNA treated with MP compared to DNA control and DNA treated with Fenton’s reagent only (lane 5).

#### 2.8.4. DNA Damage Protection by MPMC against Oxidative Cleavage of Genomic DNA

Using PCR technique, the protective properties of MPMC on genomic DNA damage induced by Fenton reaction were examined. In general, DNA damage reduces the copy number of certain genes, therefore produces lower band intensity in gel electrophoresis. Band density *MTHFR* gene of blood genomic DNA incubated with Fenton’s reagent with and without the IC_50_ of MP, MP/NabM, and MP/IHM was tested. It was found that MP/NabM and MP/IHM (lane 3 and 4, Figure 8B) prevented DNA damage, and subsequently protect *MTHFR* template cutting site. Therefore, higher PCR product band intensity in the electrophoresis gel compared to respective controls was produced (Figure 8B). These results indicated that the antioxidant activity of MPMC inhibited the production of free radicals that mediated DNA damage.

#### 2.8.5. Gene Expression

**Figure 9:**
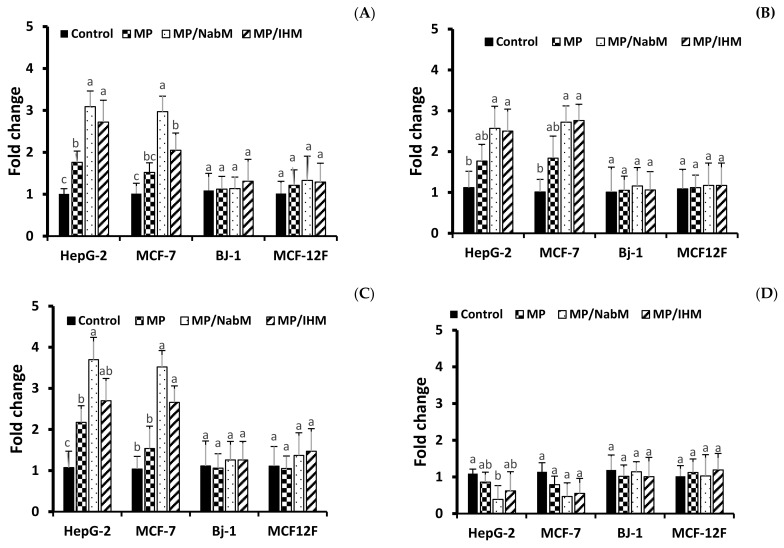
Analysis of apoptosis genes transcript in HepG-2, MCF-7, Bj-1, and MCF-12F cells treated with MP, MP/IHM, and MP/NabM. Profiling of mRNA transcript levels of key pro- (Casp3 (**A**), p53 (**B**), Bax (**C**) and anti-apoptotic Bcl-2 (**D**)). Gene expression levels were quantified after 72 h by RT-qPCR employing 18S as a housekeeping gene for normalization as detailed in the methods. Significant differences between the means of individual treatments and control were analyzed by one-side Student’s t-test. Histograms represent mean expression level as fold change SD for 3 technical and 2 biological replicas with different letters (a, b and c) are significantly different (*p*-value ≤ 0.05). MP: milk proteins concentrate; MP/NabM: milk proteins/Nabeq mucilage complex; MP/IHM: milk proteins/Isabgol husk mucilage complex.

In agreement with apoptosis protein marker obtained data, results of RT-qPCR revealed that incubation of HepG-2, MCF-7 cells with MP, MP/NabM, and MP/IHM induced a significant upregulation of pro-apoptotic mRNA markers, namely, Casp3, p53, and Bax, whereas the expression level of the anti-apoptotic marker Bcl-2 was significantly downregulated (Figure 9A–D). Caspases are actor proteins that play a vital role in apoptosis initiation and sustaining. However, upregulation of caspase 3 protein is indicative of execution of the main intrinsic pathway of apoptosis (Figure 9A,B) which is characterized by the collapse of the mitochondrial membrane with Bax-induced cytochrome c release, and activation of caspase 9 leading to the subsequent engagement of caspase 3 [42]. Upregulation of Casp3, p53, and Bax, along with reduction of Bcl-2 expression revealed that MP, MP/NabM, and MP/IHM triggered apoptosis. In this regards, upregulation of p53 stimulated expression of Bax, which in turn, will induce cytochrome c release, followed by caspase-9 and -3 activation. However, Bcl-2 as an anti-apoptosis is known to inhibit cytochrome c release [43], the treatment resulted in significant reductions in the mRNA transcript levels of the anti-apoptotic marker gene Bcl-2 due to MP/NabM and MP/IHM treatment, which facilitates unopposed Bax-induced cytochrome c release and subsequent apoptosis. In this study, MP/NabM severely mediated apoptosis compared to MP/IHM and MP as observed in (Figure 9). Observed anti-cancer effects of MP/NabM or MP/IHM proceeds via modulation of a p53-dependent apoptosis pathway, since both its protein and mRNA transcript levels were significantly up-regulated.

## 3. Materials and Methods

### 3.1. Materials

*Plantago ovata* (Isabgol) husk and the ripening fruit of *Ziziphus spina-christi* (Nabeq) were purchased from Giza, Egypt; milk protein concentrate (MP, 81.3% protein) was purchased from Fonterra company (Auckland, New Zealand). The casein and whey proteins in MPC are largely in their native and similar, micellar, form to those found in milk. All chemicals used were of analytical purity.

### 3.2. Preparation of Isabgol Husk Mucilage (IHM) and Nabeq Mucilage (NabM)

Isabgol husk mucilage (IHM) and Nabeq mucilage (NabM) were prepared according to the method described by El-Maksoud et al. [5]. Briefly, a suspension of isabgol husk was prepared by dissolving 1.2 g in 100 mL distilled water and purified with acetone solution. The resulting gel was stored in the refrigerator (4–5 °C) until used.

For NabM, the ripened Nabeq fruits were thoroughly washed with tap water to remove dirt. The fresh fruits were separated from the seeds and mixed with distilled water at a ratio of 1:10 (*w*/*v*) after being cut into small pieces. The mixture was blended using a laboratory blender (Toshiba Mixie, Japan) and allowed to stand for 12 h in the refrigerator. The mucilaginous extract was then centrifuged at 2000 rpm for 30 min and filtered through a muslin cloth. The resulting viscous soluble mucilage was precipitated with acetone. The crude mucilage obtained was stored in the refrigerator until used.

For analytical purposes, the mucilage was dried in an air oven at 40 °C and ground using ball mill MM400 (Retsch, Germany). The chemical composition of psyllium husk mucilage powder (PHMP) and Nabeq mucilage powder (NabMP) has been measured. The moisture, protein, fat, ash, crude fiber, and total carbohydrate contents were 8.49, 0.34, 0.21, 2.13, 1.53, and 87.44% for PHMP and 9.18, 4.72, 2.05, 2.69, 1.70, and 78.26% for NabMP, respectively. The resultant powder was stored at a cool temperature (4–5 °C) until further analysis.

### 3.3. Preparation of Milk Protein-Mucilage Complexes (MPMC)

Milk protein (MP) complexes with IHM and NabM were prepared according to Morr et al. with some modifications [44]. Milk protein concentrate was reconstituted in distilled water (10%). The resultant milk protein colloid was mixed with IHM and NabM at a 1:3 (*v*/*v*) ratio. The pH of the mixture was adjusted to 10 and left to stand for 10 min. After that, the pH was lowered to 3.5 using 0.1 N HCl and left for another 10 min. The pH was then adjusted to pH 4.6 using 0.5 M NaOH to collect MPMC by filtration on a filter paper. MP as well as the resultant MPMC were dried by lyophilization at −45 °C for 24 h using the Novalyphe-NL500 lyophilizer, Savant Instruments, Halprook, NY, USA.

### 3.4. Physicochemical and Functional Characteristics

#### 3.4.1. pH Measurement

The pH of resulted products at 10% solution was measured using a calibrated pH meter (HANNA, HI 902 m, Germany).

#### 3.4.2. Determination of Bulk Density (BD), Tapped Density (TD), and Carr’s Index

MPMC powder was poured into a 50 mL measuring cylinder up to 25 mL, corresponding to the untapped volume, and then tapped 5–10 times until no further change of the powder’s volume was noted corresponding to the tapped volume.

The bulk density, tapped density, and Carr’s index were determined applying the following equations [45]:

BD = mass/untapped volume(1)

TD = mass/tapped volume(2)

Carr’s index (%) = (TD − BD)/TD × 100(3)

#### 3.4.3. Water Absorption Index (WAI) and Water Solubility Index (WSI)

Water solubility and water absorption indices of MP and MPMC were examined according to the method described by Yagc and Gogus with some modifications [46]. The samples (0.5 g) were dispersed in 10 mL distilled water at 25 °C in a centrifuge tube. After standing for 30 min, with tube inversion every 5 min, the samples were centrifuged at 3500 rpm for 15 min. The filtrate was transferred onto an aluminum plate and dried for 3 hr at 105 °C. The weight of the remaining gel was recorded and WSI and WAI were determined as follows:

WAI (g/g) = Weight gain of gel/Dry weight of sample(4)

WSI% = Weight of dry solids in supernatant × 100/Dry weight of sample(5)

#### 3.4.4. Milk Protein Digestibility

Protein digestibility was determined according to Abd El-Ghany et al. [47]. Briefly, MP and MPMC samples were dispersed in distilled water at a concentration of 6.38 mg/mL, and the pH was adjusted to 8.0. One milliliter of freshly prepared enzyme stock solution (1.6 mg/mL trypsin, 3.1 mg/mL chymotrypsin, and 1.3 mg/mL ER amino peptidase1 (ERAP1) was mixed with protein suspension at 37 °C. The pH was recorded after 10 min, and enzyme activity was determined using casein as a reference. The following equation was used to calculate protein digestibility [47]:

% Digestibility = 210.46 − 18.10 (pH)(6)

### 3.5. Phenolic Fractions of PHM and NabM

Phenolic compounds of mucilage samples using HPLC analysis was conducted by the protocol discribed by Elsayed et al. [48]. Briefly, the mucilage extract was analyzed using an Agilent 1260 series HPLC system (Agilent technologies Inc., Santa Clara, CA, USA). The separation was passed using C18 column (100 mm × 4.6 mm i.d., 5 μm). The mobile phase contained of (A) water 0.2% H_3_PO_4_, (B) methanol, and (C) acetonitrile at a flow rate 0.6 mL/min. Gradient elute was as per the next scheme: 0–11 min (96% A, 2% B); 11–13 min (50% A, 25% B); 13–17 min (40% A, 30% B); 17–20.5 min (50% B, 50% C); and 20.5–30 min (96% A, 2% B). Detection wavelength (UV detector, 284 nm) was set at 284 nm. The injection size was 20 μL and the column temperature was maintained at 30 °C. Compounds were known by comparing their retention time with those from authentic standards. Calibration curves were utilized to evaluate the compound amounts.

### 3.6. Total Phenolic and Flavonoid Content of MP and MPMC

Polyphenolic content was determined by the Folin-Ciocalteu method with some modifications [5]. In brief, 2.8 mL milli-Q water containing 10 mg of MP or MPMC were mixed with 2 mL of 2% sodium carbonate, and 0.1 mL of 50% Folin-Ciocalteau reagent. After 30 min of incubation at room temperature, the absorbance of the reaction mixture was measured at 750 nm against deionized water as a blank using a spectrophotometer (Hitachi, Model 100-20). Gallic acid (GA) was used as a standard phenolic compound, and a seven points standard curve (0–200 mg/L) was constructed. The results were expressed as milligram gallic acid equivalents per gram protein (GAE/g).

Flavonoids content was determined using the aluminum chloride method as previously described by Abedelmaksoud et al. with some modifications [5]. In brief, 2.8 mL milli-Q water containing 10 mg MP or MPMC was mixed with 0.1 mL of 10% aluminum chloride hexahydrate and 0.1 mL of 1 M potassium acetate. After incubation at room temperature for 40 min, the absorbance of the reaction mixture was measured at 415 nm against deionized water as a blank. Quercetin was used as a standard utilizing a seven points standard curve (0–50 mg/L). Total flavonoid content was expressed as milligram quercetin equivalents (QE/g).

### 3.7. Antioxidant Activity of MP and MPMC

The total free radical scavenging capacity of MP and MPMC was evaluated using three different antioxidant assays: Stable 2,2-diphenyl-1-picrylhydrazyl (DPPH), Azino-bis(3-ethylbenzothiazoline-6-sulphonic acid (ABTS), and Hydroxyl radical (HS) radicals as previously described by El-Maksoud et al. [5].

### 3.8. Amino Acids Profile of MP and MPMC

Protein samples were acid hydrolyzed using 6 N HCl according to Peksa et al. [49]. An automated amino acid analyzer (AAA 400, INGOS Ltd.) was used for determining amino acids.

### 3.9. Rheological Properties

Samples were prepared as described by Barnes [50] with some modifications. The viscosity of 2.5% (*w*/*w*) solution at pH 7 was determined at 25 ± 1 °C using a Brookfield viscometer (DV-II + Pro, Rheocalc Software, Germany). Stress (σ) and viscosity (η) were plotted as a function of shear rate. The consistency index (K) and the flow behavior index (n) of the power-law model were calculated:

σ = kγ^n^ (7)

the viscosity at a given shear rate can be calculated as follows:

(8) $$\eta \;=\;\frac{\sigma }{\gamma }\;=\;\frac{k\gamma n}{\gamma }\;=\;k\gamma^{n-1}$$

The value of n equals 1 for Newtonian fluids and varies from 0 to 1 for fluids that are shear-thinning and greater than 1 for shear-thickening fluids [51].

### 3.10. Differential Scanning Calorimetry (DSC)

The thermal properties of MP and MPMC were measured using a differential scanning calorimeter (PerkinElmer, USA). Samples (4–6 mg) were placed in an aluminum plate and sealed. Each pan was then heated to 350 °C with a flow of dry nitrogen gas at 20 mL min^−1^ at a heating rate of 10 °C min^−1^. From the endothermic peak of the DSC thermogram, the denaturation onset temperature, maximum transition temperature, and degradation point were estimated [52]. The transition enthalpy value (ΔH) from the area under the endothermic peak in the DSC curve was determined, and the empty aluminum pan was used as a reference.

### 3.11. Fourier-Transform Infrared Spectroscopy (FTIR)

Infrared spectra between 600 and 3500 cm^−1^ were measured at 25 °C using an FTIR spectrophotometer (QFA Flex, USA) by the attenuated total reflection method using a resolution of 4 cm^−1^ and acquiring 10 scans per second.

### 3.12. Transmission Electron Microscopy

The microstructure of MP and MPMC was visualized using a transmission electron microscope (JEOL, JEM 1400 Flash, Japan) at 80 kv. The cryo-stage in the negative staining method was applied [53].

### 3.13. Biological Studies on MP and MPMC

#### 3.13.1. In Vitro Anticancer Activity

All cell lines used in this experiment were obtained from the American Type Culture Collection (ATCC, Manassas, VA, USA). Two human cancerous cell lines were used in this assay: mammary adenocarcinoma (MCF-7, ATCC^®^ HTB-22™) and hepatocellular carcinoma (HepG2, ATCC^®^ HB-8065™). Non-cancerous skin fibroblast BJ-1 (ATCC^®^ CRL-2522™) and epithelial breast MCF-12F (ATCC^®^ CRL-10782™) cell lines were also tested for comparison. Cell lines were grown in Dulbecco’s modified Eagle’s medium (DMEM/high glucose, HyCloneTM, USA) supplemented with 10% fetal bovine serum (FBS, Gibco, Brazil), 100 U/mL penicillin, 100 μg/mL streptomycin, and 25 ng/mL amphotericin B (Sigma, Oakville, ON, Canada) and cultured at 37 °C with 5% CO_2_ in a humidified incubator. The culture medium was replaced every other day with fresh medium. When the cells reached 70% confluency, they were detached using 0.25% Trypsin-EDTA (1X) Gibco, Canada and transferred to a new culture flask. After the second passage, the cells were trypsinized and plated in a 96-well plate at a concentration of 2 × 10^4^ cells/well. After 24 h of incubation, the medium was decanted, and the cells were washed twice with PBS then treated with 200 μL of culture medium containing different concentrations of protein samples (1, 5, 10, and 20 μg/mL). Doxorubicin HCl (4 μg/mL) was used as positive control, while cells treated with unsupplemented medium were taken as negative control. The plates were incubated at 37 °C for 24 h and the anti-cancer activity of the samples was determined using neutral red uptake assay according to Repetto et al. [54]. Briefly, after the incubation period, the medium was replaced with 150 μL of neutral red dye (100 mg/mL) dissolved in serum-free medium, and the cells were incubated at 37 °C for 3 h. Next, the cells were rinsed with PBS, and 150 μL of elution medium (EtOH/AcCOOH, 50:1) was added followed by gentle shaking for 10 min to ensure complete dissolution. Absorbance was measured at 540 nm using a microtiter plate reader (BioTek, 96 Well Plates, ELX808, USA).

(9) $$\mathrm{Cytotoxicity}\;(\%\;\mathrm{inhibition})\;=\;100-(\frac{\left(\mathrm{Ac}\;-\;\mathrm{As}\right)}{\mathrm{Ac}}\;\times \;100)$$

where Ac is the absorbance of the control and As is the absorbance of the sample. Percentage cell death presented as cytotoxicity (%) and the concentration that kills 50% of cells (IC_50_) was calculated for each sample.

#### 3.13.2. Determination of p53, Bax, Caspase-3, and Bcl-2 Proteins Level

The cellular levels of key apoptosis protein markers were determined after 24 h post-treatment with the IC_50_ of MP and MPMC. Briefly, HepG-2, MCF-7, Bj-1, and MCF-12F cells were seeded at 5 × 10^6^ cells/well in 6-well plates. After 24 h of incubation with the samples at 37 °C, cells were collected and lysed then centrifuged at 10,000 rpm for 20 min at 4 °C. Protein concentration was quantified in the supernatant by Bradford assay [55]. Caspase-3 activity was analyzed using a colorimetric assay kit (Abcam, ab39401) according to the manufacturer’s instructions [56]. In brief, 10 mg protein was added to 50 µL of Caspase substrate and the final volume was adjusted to 200 µL using the reaction buffer at 37 °C, and the mixture was incubated for 1 h in the dark followed by measuring Caspase-3 concentration at 405 nm.

The level of pro-apoptotic marker p53, associated X (Bax), and anti-apoptotic marker B-cell lymphoma-2 (Bcl-2) were determined in cell lysate using Enzyme-Linked Immunosorbent Assay Simple Step ELISA^®^ according to the manufacturer’s instructions (ab207225, ab119506, and ab199080; Abcam, USA), respectively [57,58].

#### 3.13.3. DNA Damage Induced by Oxidative Stress Protection

DNA damage assay was carried out as previously described by Leba et al. [59] with slight modification. In brief, 3 μL of Ribonuclase inhibitor plasmid RNH1 (NM_203387) Human Tagged ORF Clone 10 μg/μL were mixed with Fenton’s reagent (5 mM of H_2_O_2_ and 0.3 mM of FeSO_4_ and 0.6 mM of EDTA), and the final volume was adjusted to 25 μL using phosphate buffer (H_2_PO_4_, 8.3 mM, pH 7.4). Then, the solution was incubated for 20 min at 37 °C. To assess the antioxidant protection capacity of MP and MPMC against DNA damage induced by Fenton’s reagent, 5 μg/mL MP/NabM, MP/IHM, and MP were added before incubation. RNH1 plasmid DNA (3 μL, 10 μg/μL) was used as DNA protection control.

#### 3.13.4. PCR-Based Genomic DNA Damage Assay

Homo sapiens methylenetetrahydrofolate reductase (*MTHFR*) gene was amplified using PCR from blood genomic previously extracted from healthy blood samples using a standard DNA extraction kit [60]. DNA was incubated for 10 min with or without Fenton’s reagent supplemented with MP or MPMC. A 198-bp fragment was amplified using forward 5′-TGAAGGAGAAGGTGTCTGCGGGA-3′ and reverse 5′-AGGACGGTGCGGTGAGAGTG -3′ primers. Using 50 ng of treated DNA, PCR amplification was carried out following next thermocycle (94 °C for 5 min, followed by 25 cycles of denaturation, 30 s at 94 °C; annealing, 30 s at 60 °C; and extension, 30 s at 72 °C) for 25 cycles and 1 final extension cycle at 72 °C for 5 min. The PCR reaction samples were applied for agarose gel electrophoresis (2%).

#### 3.13.5. Gene Expression Profiling by RT-qPCR

Analyses of gene expression of key pro-and anti-apoptotic marker genes were conducted with HepG-2, MCF-7, Bj-1 and MCF-12F cells exposed to IC50 obtained data (Table 3) for 72 h. Briefly, total RNA was extracted using the Total RNA Purification Kit (QIAGEN., Thorold, ON, Canada) according to manufacturer’s instructions. cDNA synthesis was carried out as previously described by Aboul-Soud et al. [61]. Amplification programs and PCR amplicon specificity were performed and assessed by the use of a Rotor-Gene Q 5-Plex HRM thermal cycler (Qiagen, Germany) with QuantiTect SYBR-Green PCR Kit (Qiagen, Germany) as previously documented following standard protocols [61]. The following primers were used: Hs_P53_1_SG QuantiTect Primer Assay (QT00060235); Hs_CASP3_1_SG Quan-tiTect Primer Assay (QT00023947); B-cell lymphoma 2 Hs_BCL2_1_SG QuantiTect Primer Assay (QT00025011); Bcl-2-like protein Hs_BAX_1_SG QuantiTect Primer Assay (QT00031192); and 18S rDNA house-keeping (HK) gene Hs_RRN18S_1_SGQuantiTect Primer Assay (QT00199367). PCR thermal cycling program and gene expression analysis to determine the fold-change relative to the 18S gene were essentially performed as previously reported [61].

### 3.14. Statistical Analysis

The obtained results were expressed as mean ± standard deviation. Statistics were achieved using SPSS software 11.5 (SPSS, Inc. © Chicago, IL, USA). Duncan’s multiple range test was used to determine the significant differences among all samples, and differences were considered significant at *p* < 0.05.

## 4. Conclusions

In conclusion, this study revealed that mixing of milk protein with polysaccharides through the differentiation of solution pH is a suitable technique for complexation. The resulting milk protein mucilage complexes (MPMC) have significantly higher phenolic and flavonoids contents than the milk protein (MP). DSC results showed that complexation of MP with polysaccharides significantly increased the thermal stability with a shift in denaturation temperature. FTIR analyses were performed to identify the functional groups, and the flow behavior of the prepared complexes was investigated. The WSI and WAI of milk protein were increased by complexation with NabM or IHM. TEM was used to visualize the microstructure of the prepared complexes. On the other hand, the resulting milk protein complexes with isabgol husk mucilage (IHM) or Nabeq mucilage (NabM) are expected to outperform the native milk proteins in the antioxidant and anticancer effect. In summary, the MPMC expressed unique anticancer properties against model breast cancer and hepatic carcinoma cell lines (MCF7 and HEPG2, respectively) as proven by a neutral red uptake assay.

MPMC enhanced both antioxidant and anti-proliferation activity. Caspases are key regulator proteins that are implicated in apoptosis induction. Upregulation of caspase 3 protein and mRNA transcripts (Figure 8A,B) control other proteins in the pathway of apoptosis, which is characterized by the collapse of the mitochondrial membrane with Bax-induced cytochrome c release and activation of caspase 9 leading to the subsequent engagement of caspase 3. Up-regulation of Casp3, p53, and Bax, along with reduction of Bcl-2 expression revealed that MPMC induced apoptosis induction, where upregulation of p53 will stimulate expression of Bax, which, in turn, will induce cytochrome c release, followed by caspase-9 and -3 activation. Moreover, Bcl-2 is known to inhibit cytochrome c release. In our study, MPMC -induced Bcl-2 downregulation was shown to facilitate unopposed Bax-induced cytochrome c release and subsequent apoptosis.

The anticancer effect of protein-polysaccharide complexes provides further enlightenment on the applicability of these complexes to be used in the development of affordable and efficient cancer therapies with minimal side effects.

## Figures and Tables

**Table 1:** Physicochemical and functional characteristics of MP, NabM, IHM, and MPMC.

	IHM	NabM	MP	MP/IHM	MP/NabM
pH	7.79 ± 0.21 ^a^	6.42 ± 0.61 ^b^	4.7 ± 0.21 ^d^	5.1 ± 0.1 ^c^	4.6 ± 0.22 ^d^
Bulk density (g/cm^3^)	0.34 ± 0.04 ^c^	1.25 ± 0.07 ^a^	1.18 ± 0.05 ^a^	0.55 ± 0.01 ^b^	1.23 ± 0.01 ^a^
True density (g/cm^3^)	0.53 ± 0.02 ^e^	1.42 ± 0.04 ^a^	1.25 ± 0.03 ^c^	0.91 ± 0.01 ^d^	1.29 ± 0.01 ^b^
Carr’s Index (%)	57.58 ± 0.14 ^a^	53.97 ± 0.11 ^b^	30.6 ± 0.10 ^e^	39.56 ± 0.12 ^c^	33.65 ± 0.18 ^d^
WSI (%)	71.35 ± 1.52 ^a^	33.52 ± 0.24 ^c^	15.87 ± 1.04 ^e^	36.67 ± 0.16 ^b^	21.42 ± 0.13 ^d^
WAI (g/g)	21.42 ± 0.12 ^a^	8.13 ± 0.15 ^c^	3.54 ± 0.04 ^e^	16.63 ± 0.53 ^b^	5.50 ± 0.29 ^d^
Protein digestibility (%)			79.44 ± 0.5 ^a^	81.63 ± 1.22 ^a^	75.63 ± 1.22 ^b^

NabM: Nabeq mucilage, IHM; Isabgol husk mucilage, MP: milk proteins concentrate, MP/IHM: milk proteins/Isabgol husk mucilage complex, MP/NabM: milk proteins/Nabeq mucilage complex, WSI: water solubility index; WAI: water absorption index. Values are means ± SD, n = 3. Different lowercase superscripts (a–e) in the same row indicate significant differences (*p* ≤ 0.05).

**Table 2:** Differential scanning calorimetry (DSC) parameters of MP and MPMC *.

Samples	MP	MP/IHM	MP/NabM
Denaturation onset (°C)	50.01	138.12	62.89
Protein denaturation (°C)	101.44	148.24	121.4
Degradation degree (°C)	302.3	-	-
ΔH (J/g)	98.63	553.08	121.82

* ΔH J/g: enthalpies joule/gram protein. MP: milk proteins concentrate, MP/IHM: milk proteins/Isabgol husk mucilage complex and MP/NabM: milk proteins/Nabeq mucilage complex.

**Table 3:** Cancer cytotoxic IC_50_ of MP and MPMC on HEPG-2, MCF-7, MCF7-12F, and Bj-1 cells.

Cell Lines	IC_50_ µg/mL			
	MP	MP/NabM	MP/IHM	DOX
HepG2	18.2 ± 1.86 ^a^	5.13 ± 0.31 ^b^	8.89 ± 0.15 ^b^	14.87 ± 0.19 ^c^
MCF-7	22.5 ± 2.75 ^a^	10.07 ± 0.72 ^c^	12.3 ± 0.018 ^b^	0.74 ± 1.18 ^b^
MCF7-12F	198.4 ± 6.23 ^c^	256.47 ± 7.25 ^a^	215.32 ± 4.34 ^b^	24.16 ± 7.23 ^b^
Bj-1	203.34 ± 2.45 ^c^	238.56 ± 3.54 ^a^	236.67 ± 4.78 ^b^	36.23 ± 5.64 ^c^

MP: milk proteins concentrate; MP/NabM: milk proteins/Nabeq mucilage complex; MP/IHM: milk proteins/Isabgol husk mucilage complex; DOX: Doxorubcin positive control. The IC_50_ was calculated from the serial concentration and represented in µg/mL. Values are means ± SD (n = 3). Measurements with different letters (a, b, and c) are significantly different (*p* < 0.05).

## Data Availability

All data is included within the manuscript.

## Supplementary Materials

The following are available online, Figure S1: The apparent viscosity of MP, IHM, NabM, and MPMC. MP: milk protein, IHM; isabgol husk mucilage, NabM: Nabeq mucilage, MP/IHM: milk proteins/isabgol husk mucilage complex and MP/NabM: milk proteins/Nabeq mucilage complex; Figure S2: Shear stress vs. shear rate curves of BTMP: buffalo total milk protein, NabM: Nabeq mucilage, IHM: isabgol husk mucilage, BTMP/NabM: buffalo total milk proteins/Nabeq mucilage complex, BTMP/IHM: buffalo total milk proteins/isabgol husk mucilage complex; Figure S3: Differential scanning calorimetry thermograms of: (a) MP: milk proteins concentrate, IHM: Isabgol husk mucilage; (b) MP/IHM: milk proteins/Isabgol husk mucilage complex; (c) MP: milk protein, NabM: Nabeq mucilage and MP/NabM: milk proteins/Nabeq mucilage complex; Table S1: Parameters of the power-law equation of MP and MPMC.
