# Correction: El-Maksoud et al. Nano Milk Protein-Mucilage Complexes: Characterization and Anticancer Effect. *Molecules* 2021, *26*, 6372

**DOI:** 10.3390/molecules31142400

**Published:** 2026-07-08

**Authors:** Ahmed Ali Abd El-Maksoud, Amal I. A. Makhlouf, Ammar B. Altemimi, Ismail H. Abd El-Ghany, Amr Nassrallah, Francesco Cacciola, Tarek Gamal Abedelmaksoud

**Affiliations:** 1 Dairy Science Department, Faculty of Agriculture, Cairo University, Giza 12613, Egypt; 2 Pharmaceutics and Industrial Pharmacy Department, Faculty of Pharmacy, Cairo University, Cairo 12411, Egypt; 3 Food Science Department, College of Agriculture, University of Basrah, Basrah 61004, Iraq; 4 Biochemistry Department, Faculty of Agriculture, Cairo University, Giza 12613, Egypt; 5 Messina Institute of Technology c/o Department of Chemical, Biological, Pharmaceutical and Environmental Sciences, University of Messina, Viale G. Palatucci 13, 98168 Messina, Italy; 6 Food Science Department, Faculty of Agriculture, Cairo University, Giza 12613, Egypt

Figure Legend

In the original publication [1], there was a mistake in Figure 5D. The relationship between panels Figure 5D1 and Figure 5D2 was not sufficiently clarified in the published figure legend. Figure 5D1 represents an enlarged view of a selected region from Figure 5D2. To improve clarity, the legend has been revised and the corresponding source area in Figure 5D2 has been indicated. The correct legend appears below.

**Figure 5:**
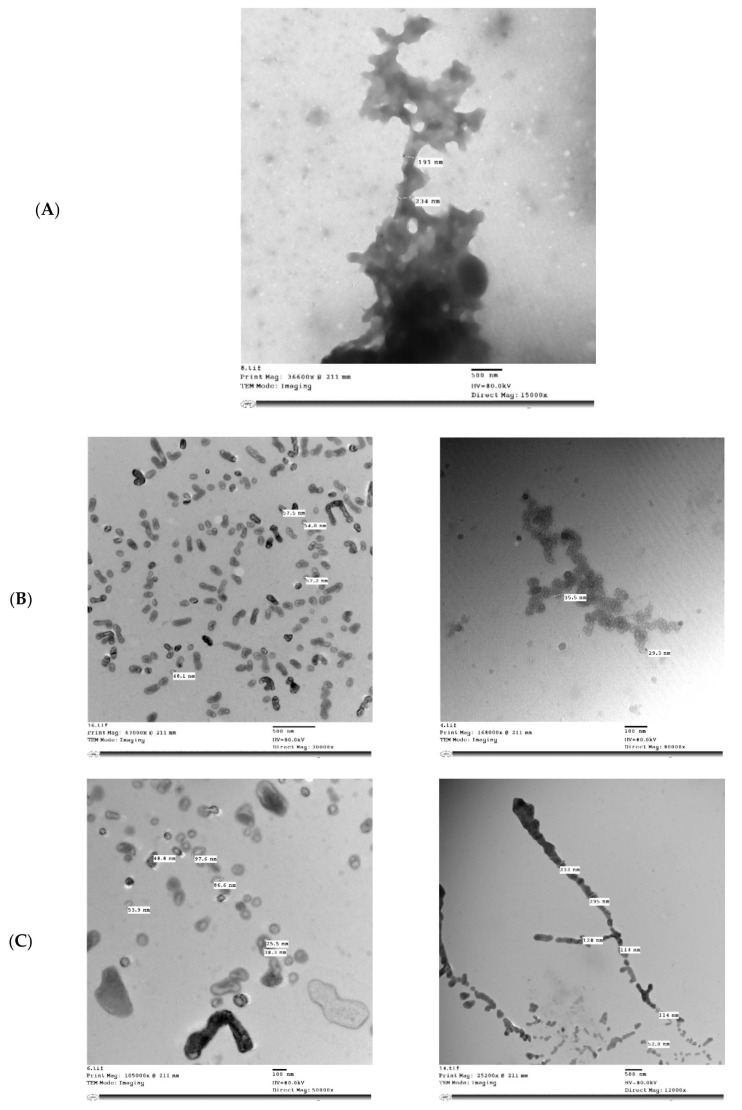

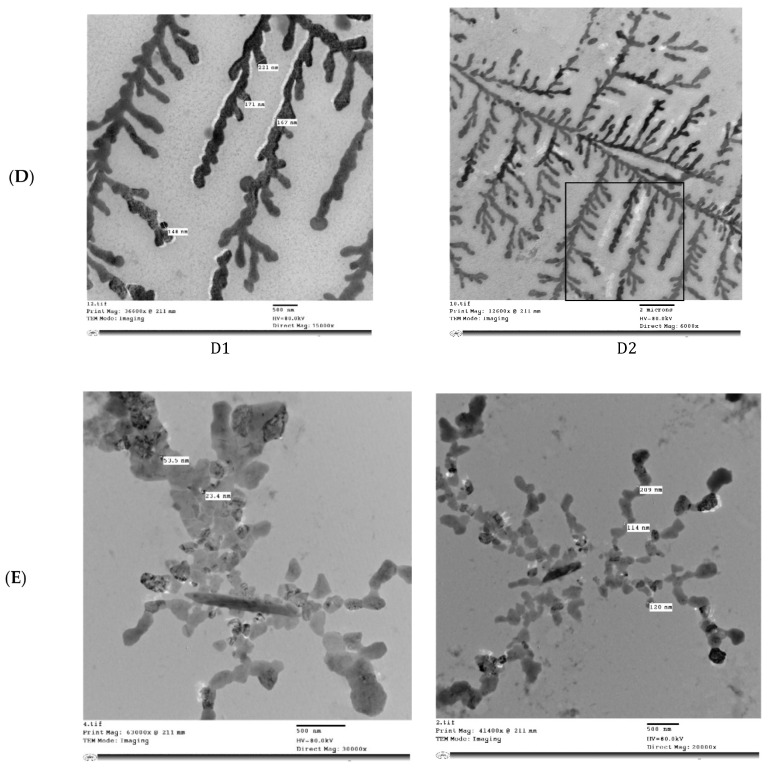
Transmission electron microscopy images of (**A**) MP, (**B**) IHM, (**C**) NabM, (**D**) MP/IHM, and (**E**) MP/NabM. Panel D2 shows the original micrograph; the boxed region indicates the area enlarged in panel D1. MP: milk protein concentrate; MP/NabM: milk protein–Nabeq mucilage complex; MP/IHM: milk protein–Isabgol husk mucilage complex.

Error in Figure 7. Concerns were raised regarding the presentation of certain micrographs in Figure 7. Figure 7 was included as a representative illustration of morphological changes and was not essential to the quantitative analyses or to the conclusions of the study. Following evaluation by the two Academic Editors and in the interest of maintaining the highest standards of scientific transparency and integrity, Figure 7 has been removed from the article.

The authors emphasize that Figure 7 served only as a representative morphological illustration and that the quantitative findings and conclusions of the study remain supported by the remaining experimental data and analyses presented in the article.

The authors apologize for any inconvenience caused and state that the scientific conclusions are unaffected. This correction was approved by the two Academic Editors. The original publication has also been updated.
