# Supplementary material for: Nano Milk Protein-Mucilage Complexes: Characterization and Anticancer Effect
Source: Molecules. 2021 Oct 21;26(21):6372. doi: 10.3390/molecules26216372 (PMC8588565; doi:10.3390/molecules26216372)
Supplement: Supplementary file 1 [file molecules-26-06372-s001.zip › molecules-1338099-supplementary.pdf]

molecules

Supplementary Data

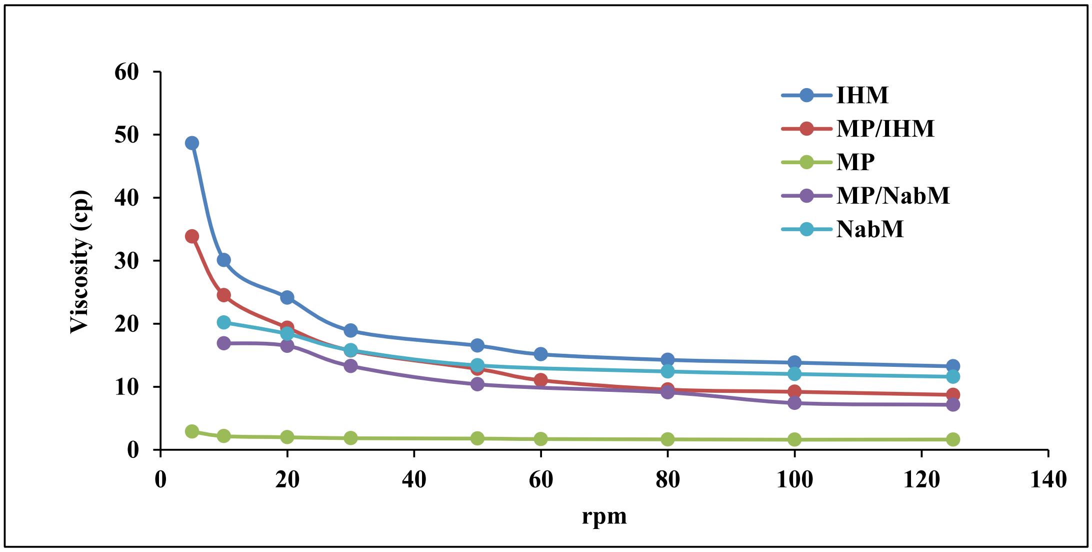

Figure S1. The apparent viscosity of MP, IHM, NabM and MPMC: MP: milk protein, IHM; isabgol husk mucilage, NabM: Nabeq mucilage, MP/IHM: milk proteins/isabgol husk mucilage complex and MP/NabM: milk proteins/ Nabeq mucilage complex

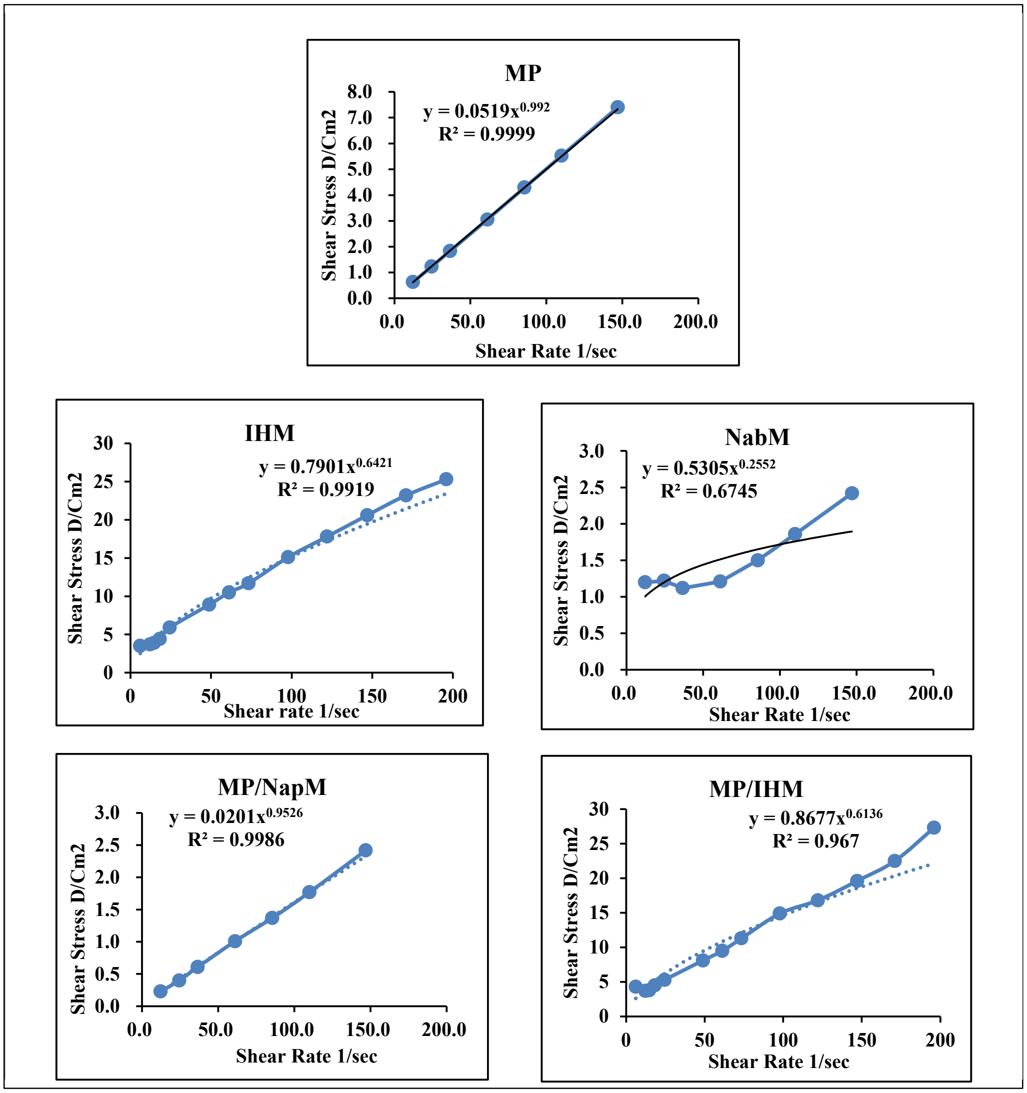

Figure S2. Shear stress vs shear rate curves of BTMP: buffalo total milk protein, NabM: Nabeq mucilage, IHM: isabgol husk mucilage, BTMP/NabM: buffalo total milk proteins/ Nabeq mucilage complex, BTMP/IHM: buffalo total milk proteins/isabgol husk mucilage complex

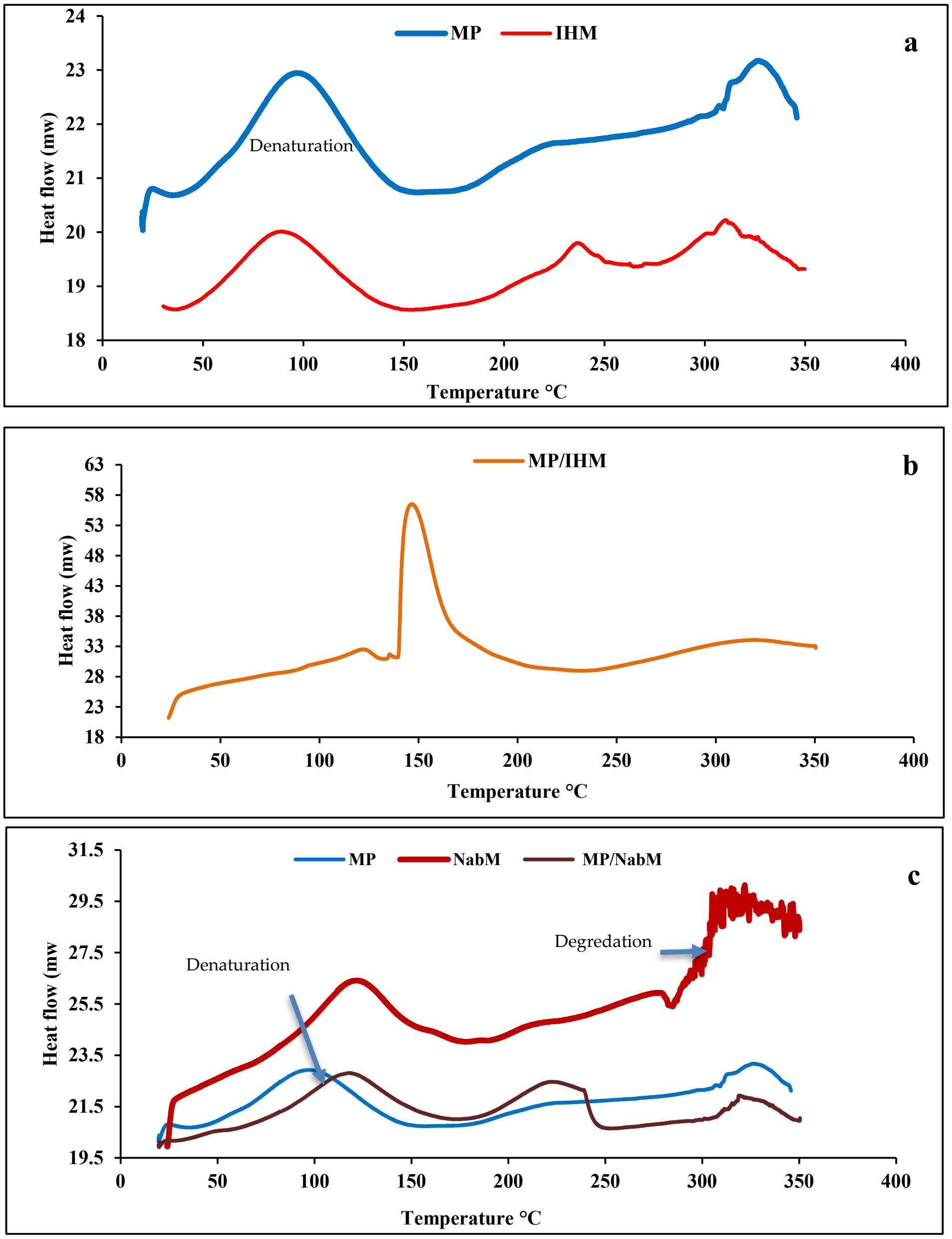

Figure S3. Differential scanning calorimetry thermograms of: a) MP: milk protein, IHM: isabgol husk mucilage; b) MP/IHM: milk proteins/isabgol husk mucilage complex; c) MP: milk protein, NabM: Nabeq mucilage and MP/NabM: milk proteins/Nabeq mucilage complex

Table S1. Parameters of the power law equation of MP and MPMC*.

Samples	IHM	NabM	MP	MP/IHM	MP/NabM
**K (Pa.s^n)**	0.79	0.53	0.053	0.87	0.027
**n**	0.64	0.25	0.992	0.61	0.95
**R^2**	0.97	0.62	0.99	0.93	0.99

*K: consistency index; n: flow behavior index; R2: R-squared statistic; NabM: Nabeq mucilage; IHM: isabgol husk mucilage; MP: milk protein; MP/NabM: milk proteins/Nabeq mucilage complex; MP/IHM: milk proteins/isabgol husk mucilage complex.
